# Geographic areas with the highest concentration of traffic accidents in San Salvador, El Salvador: a spatial analysis of the 2014-2018 period

**DOI:** 10.17843/rpmesp.2023.404.12963

**Published:** 2023-12-18

**Authors:** Roberto Mejía, Edgar Quinteros, Alexandre Ribó Arnau

**Affiliations:** 1 Instituto Nacional de Salud, San Salvador, El Salvador. Instituto Nacional de Salud San Salvador El Salvador; 2 Departament d'ensenyament, Generalitat de Catalunya, España. Departament d'ensenyament Generalitat de Catalunya España

**Keywords:** Traffic Accidents, Accident Prevention, Geographic Information Systems (GIS), El Salvador, Central America

## Abstract

**Objective.:**

This study aimed to identify the areas with the highest concentration of traffic accidents and injuries in the San Salvador Metropolitan Area (SSMA).

**Materials and methods.:**

Traffic accidents were analyzed spatially by point location and by the sum of events in areas of 200 m2. The point location was analyzed by “nearest neighbor analysis”, while the areas with the sum of traffic accidents were analyzed by Getis-Ord Gi* to obtain the hot spots. The resulting hot spots with the highest concentration of traffic accidents in the SSMA were evaluated in the field using an observation form to collect data on infrastructure and road safety characteristics.

**Results.:**

Five areas with the highest number of traffic accidents and injuries, mainly containing primary roads, were identified by analyzing 8191 traffic accidents reported between 2014-2018.

**Conclusion.:**

The sites with the highest concentration of traffic accidents and injuries were characterized by considerably damaged road infrastructure and the lack of safety systems for drivers and pedestrians. The spatial analysis of traffic accidents and injuries can contribute to improve surveillance and road safety in the SSMA.

## INTRODUCTION

Road traffic accidents are a serious public health problem worldwide. According to the World Health Organization (WHO), road traffic fatalities increased from 1.15 to 1.35 million between 2000 and 2016 ^1^. In Latin America, 154 997 fatalities were reported in 2016, representing 11% of deaths worldwide ^2^. The Latin American countries most affected by road traffic accidents are El Salvador, Brazil, Venezuela, Colombia, and Ecuador ^3^^,^^4^.

In El Salvador, traffic accidents represent a major public health problem. In the 2014-2018 period, reports showed 38,531 injured persons and a total of 3573 fatalities ^5^. Each year an average of US$15 million is invested in care for victims of traffic accidents ^6^. The city of San Salvador, where the highest number of traffic accidents in Latin America is reported ^3^, is located in a large part of the San Salvador Metropolitan Area (San Salvador Metropolitan Area, SSMA). During 2019, 51.5% of the country’s traffic accidents occurred in the SSMA ^5^.

Some of the roads in SSMA have been built in an unplanned manner and under certain social conditions that determined their current design and condition. SSMA has small parts of isolated urban settlements that lack infrastructure and basic services. These settlements began in the mid-1950s as a result of unplanned growth and the lack of a land-use planning law ^7^^,^^8^. This was compounded by the migratory flow from the interior of the country to SSMA during the 1980s as a result of the armed conflict in El Salvador ^7^.

Geographic analysis is one of several analytical alternatives for understanding the problem of traffic accidents. Its contribution is based on identifying spatial distribution patterns of traffic accidents and the environmental factors that may be related. In recent decades, Geographic Information Systems (GIS) have been used to analyze the spatial distribution patterns of traffic accidents hotspots in urban areas ^9^^,^^10^. GIS, a set of tools and components that allow information management through the use of spatial technology, have been widely used in public health. Particularly in Latin America, they have been useful in identifying the spatial distribution of road traffic injuries and mortality in areas of high vehicular traffic ^11^^,^^12^. Other studies have also estimated the vulnerability of road users by analyzing the spatial density of collisions and their characteristics for the occurrence of traffic accidents ^13^^,^^14^.

There is an increasing integration of geospatial information in health surveillance databases in El Salvador, but geographic studies on areas with the highest concurrence of traffic accidents are not yet available. The aim of this study is to identify the geographic areas of highest concentration of road traffic accidents and injuries in the SSMA in the period of 2014-2018. The results contribute to the knowledge of territorial patterns with more accidents through the application of analysis tools available in GIS.

**KEY MESSAGES**

Motivation for the study: Traffic accidents are a serious public health problem in El Salvador, especially in the Metropolitan Area of San Salvador (AMSS). There is no scientific evidence of critical areas in the SSMA and the country.

Main findings: Five geographic areas were identified in which a large number of traffic accidents and injuries were registered. They are characterized by road infrastructure with considerable damage, lack of safety systems, poor road signage, visual pollution, and lack of lighting.

Implications: Identification of critical areas for traffic accidents can contribute to the creation of public policies for prevention.

## MATERIALS AND METHODS

### Study design

This is an analytical cross-sectional study with information on traffic accidents and injuries in the SSMA. We used information from the Emergency Medical System (MES), a nationwide emergency care coordination network of the Ministry of Health (MINSAL) that has an immediate response system.

### Study area

**Figure 1:**
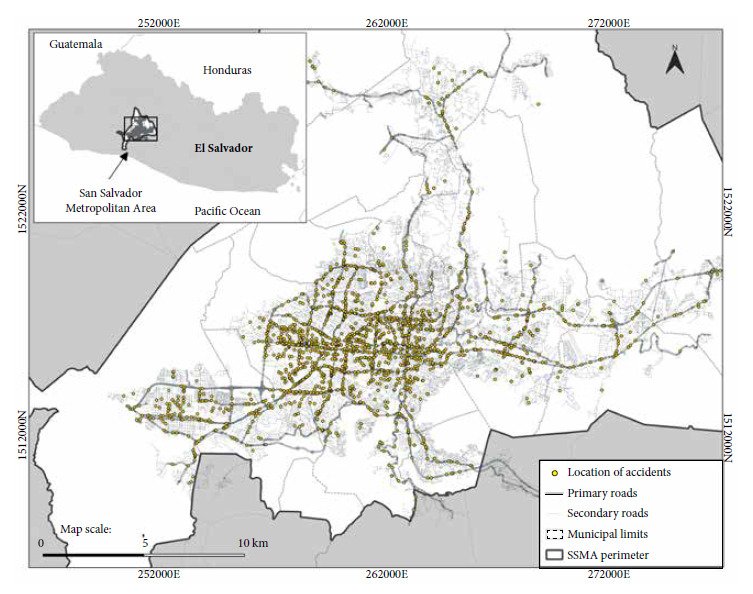
Location of the SSMA and spatial distribution of traffic accidents during 2014-2018.

The SSMA is a conurbation located over 14 municipalities (Figure 1), with a territorial extension of 610.84 km^2^, a population of 1,693,186 inhabitants and a population density of 2772 h/km^2 (^^15^.

### Data

The data on traffic accidents and injuries occurring in the SSMA correspond to the period 2014-2018. The data and permissions were requested in writing to MES who provided an Excel file. The MES registry does not include cases of people who died due to traffic accidents, these cases are attended by the National Civil Police and Institute of Legal Medicine, therefore, deaths due to traffic accidents have not been analyzed in this study.

The 15,497 records provided by MES correspond to care provided in the country’s 262 municipalities. Only 8191 traffic accidents that occurred in the SSMA were selected for this study. Information on the spatial location where the traffic accidents occurred was obtained using the global positioning system (GPS) or manually using web maps. Due to location errors in the coordinates, it was necessary to make an adjustment by verifying the postal addresses of the MES care registry and using the spatial object movement tool available in GIS.

### Geocoding of data

Previously, we carried out a geocoding process for the spatial analysis, which consisted of transforming the study variables into geographic locations. A layer of points of traffic accidents that occurred in the SSMA was created from the MES data. The analysis also required the use of cartography referring to roads and national geographic delimitation that was obtained from the National Registration Center. Using the road layer, a new selection was created only of the roads that correspond to the SSMA. Finally, with the territorial extension of the SSMA, we created a 200 m^2^ grid containing the sum of traffic accidents, a mapping technique known as observation counting, similar to that used by Adams *et al*. ^16^.

### Study variables

We selected and reclassified the variables originally contained in the SEM information. Variables with repeated information, typing errors or empty fields were eliminated. The variables “Year”, “Weekday”, “Weekend” and “Holidays” were reclassified from the information of the "demand date" variable. The variables “Collision” and “Crash” were defined from the information of the “type of demand” variable. Finally, the reclassified variables “injured”, “motorcyclists” and “run over” were obtained from the “literal motive” variable which contained details of the type of vehicle, injured riding in the vehicle or run over in the traffic accident. The description of the study variables is shown in Supplementary Table 1.

### Inspection of sites with the highest concentration of accidents in the SSMA

The areas identified as hot spots were visited in order to observe the road infrastructure and road safety aspects. The field visits were conducted by the study researchers, with the support of epidemiology resident physicians. All the information was collected through a self-created form in KoBo ToolBox ^17^, which is available at https://ee.kobotoolbox.org/eTC7EiC2. Some of the terms used for road system characteristics were based on the provisions of the Transportation Law of El Salvador, scientific articles, and terminology defined by international organizations ^18^^-^^20^. To validate the form, the authors of this study conducted a pilot test at three critical points on the outskirts of the SSMA on a weekday during peak traffic hours.

### Statistical analysis

A descriptive analysis was performed to identify the frequency of traffic accidents, number of people injured, type of collision, distribution by month and year, and road safety infrastructure characteristics of the sites with the highest concentration of traffic accidents. The distribution of the population was tested using the Kolmogorov-Smirnov test and a comparison of medians of the number of accidents and injured persons during weekdays (Monday to Friday), weekends (Saturday and Sunday) and holidays (23 days in the year) using the Kruskal Wallis test.

### Geographic analysis

We used ArcGIS Desktop version 10.1 and QGIS version 3.16 for data analysis and visualization design, respectively. The geographic analysis of traffic accidents was performed at the point level and by area. The distribution of traffic accidents in the road network was identified at the point level using the non-parametric method called Nearest Neighbor Analysis ^21^ to measure the average distance between each point.

**Figure 2:**
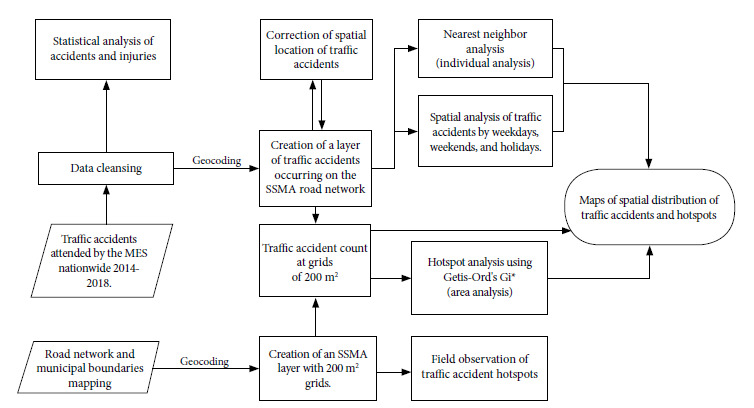
Flow chart of data processing and analysis of traffic accidents in the San Salvador Metropolitan Area**.**

At the area level, hot spots of traffic accidents were determined from 200 m^2^ polygons. Getis-Ord’s Gi* statistical tool ^22^^)^ was used to obtain the statistical significance of each grid. The values resulting from the Getis-Ord Gi* analysis show hot spots with high or low values of traffic accidents. The hot spot analysis can result in three results, high, medium or low clustering. For each of these groupings, the software presents different Z-score values (standard deviation values), p-values and confidence values. Z-score values > 1.65 with a p < 0.1 have a 90% confidence (low clustering); Z-score values > 1.96 with a p < 0.05 have a 95% confidence value (medium clustering) and Z-score values >2.58 with a p < 0.01 have a 99% confidence value (high clustering). Figure 2 presents a flow chart of the data processing and analysis of traffic accidents in the SSMA.

### Ethical Aspects

This study did not involve the analysis of human samples; however, it was reviewed and approved by the Ethics Committee of the National Institute of Health. The information was treated confidentially among the authors of this study, respecting all aspects of good clinical practice and research ethics.

## RESULTS

### Statistical analysis

A total of 8191 traffic accidents were recorded, in which 8996 people were injured, 75.6% of whom were men. The age of those affected ranged from 0 to 98 years, with an average of 33.6 years (standard deviation: 14.4). More than 60% of the traffic accidents involved persons between 20 and 39 years of age. At least one motorcyclist was involved in 30.5% of the traffic accidents. At least one person was hit by a car in 40.9% of the traffic accidents; 8.7% of traffic accidents were caused by a crash or collision. The cumulative incidence rate for the period under study is 531.3 × 100,000 population.

### Geographic analysis

*Traffic accident distribution pattern*

**Figure 3:**
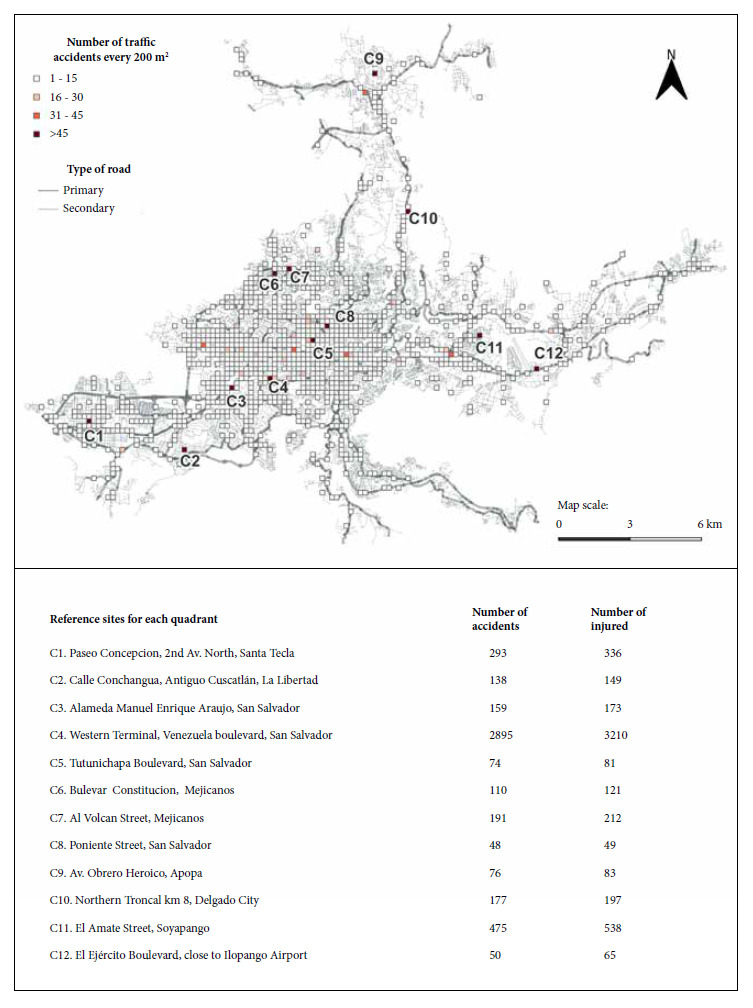
Traffic accident count in the San Salvador Metropolitan Area per 200 m².

The “nearest neighbor analysis” identified a pattern of clustered points, with an average nearest neighbor index of 0.13, an average observed distance of 17.40 m, and an average expected distance of 130.90 m. The highest number of traffic accidents and injuries is concentrated in 12 grids identified in Figure 3 with a code (a letter C and a number). The highest number of traffic accidents (2895) and injuries (3210) were recorded in the C4 area. In second place, C11, where 474 traffic accidents and 538 injuries were recorded. C1 is in third place, which is located in the west of the SSMA, where 297 traffic accidents and 340 injuries were reported. Between 1 and 15 traffic accidents occurred in most of the grids.

*Hotspots of traffic accidents*

**Figure 4:**
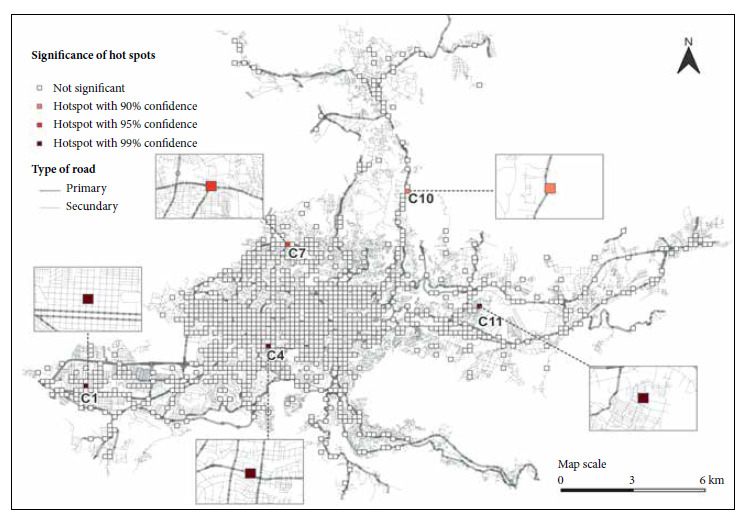
Hotspots of traffic accidents in the San Salvador Metropolitan Area**.**

Figure 4 shows the results from the hotspot analysis. A total of five hotspots were identified, totaling 4031 traffic accidents resulting in 4483 injuries during the study period. The grid with the highest density was C4 with a Z-score value of 31.3 (p<0.001). The second grid with a high density of traffic accidents was C11 (Z-score 5.06; p<0.001), followed by C1 (Z-score 3.09; p<0.001), C7 (Z-score of 1.98; p=0.04) and finally C10 with a Z-score of 1.83; p= 0.06.

*Spatial distribution of traffic accidents and injuries according to working days, weekends or holidays*

**Figure 5:**
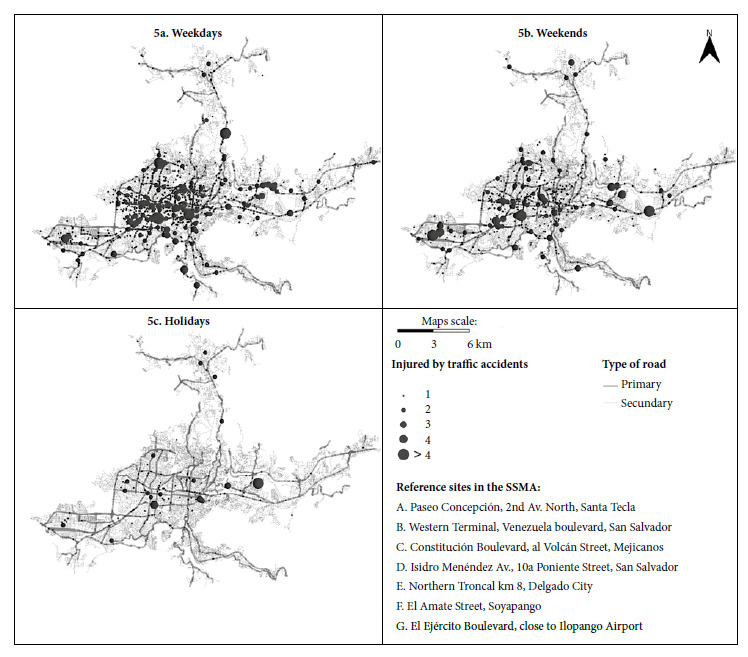
Spatial distribution of traffic accidents during weekdays (5a), weekends (5b) and holidays (5c).

A higher number of traffic accidents (5626) were recorded during work days (Figure 5a) compared to weekends (2174) (Figure 5b) and holidays (391) (Figure 5c). Traffic accidents recorded on weekdays are distributed in 1542 points located towards the northwest of the SSMA. On weekdays, we recorded 56 points, in which 11 or more traffic accidents occurred (median=18, Q3=84, Q1=14). On weekends, 11 or more traffic accidents occurred at 28 points (median=31, Q3=61, Q1=13) and on holidays there were 3 points at which 11 or more traffic accidents occurred (median=25, Q3=149, Q1=17) (Supplementary Material 1). We found a significant difference (p<0.01) between the number of traffic accidents occurring on weekdays, weekends and holidays when we compared medians.

A total of 6098 injuries were reported during weekdays, with a median of 4.69. On the other hand, 2457 injuries were reported on weekends, with a daily median of 5.01 injuries per weekend. Furthermore, 441 people were injured in traffic accidents during holidays, with a daily median of 3.83 injuries. However, through a median comparison analysis, we found a statistically significant difference (p<0.01) between the number of people injured in traffic accidents occurring on weekdays, weekends and holidays.

### Characterization of the sites with the highest occurrence of traffic accidents

Supplementary material 2 shows *in situ* information on the road characteristics of the statistically significant grids (hotspots) in the SSMA. C11 was inspected due to social insecurity. Three of the grids (C4, C7 and C10) have mostly primary roads, the roads are built on asphalt in C1 and C4 and on cement in C10 and C7. The widest roads are found in C12 (24 m) and C10 (40 m). C1 and C7 have narrower roads at 14 m and 16 m, respectively.

C1 was the only area with no structural damage, and only the C10 road has a vehicle containment system, but it was partially damaged. In terms of road signs, there is a predominance of preventive vertical road signs (stop signs), but they are in poor condition. There are illegal bus stops in three of the grids (C4, C7, C10), and there are a large number of billboards in C1, C4 and C7. Economically active zones were observed in all grids. Pedestrian infrastructure such as sidewalks was found in grids C1, C4 and C7, C10 had a shoulder and C4 and C10 have walkways.

C4 is one of the main bus stations in the capital, with a high pedestrian and vehicular flow. The sidewalks around the bus station are used by informal businesses and as a parking lot for vehicles. In addition, some vehicles were parked on the road, reducing vehicular traffic to one lane in each direction.

## DISCUSSION

Our study identified five hotspots where the highest number of traffic accidents and injuries were found in the SSMA. These hotspots are characterized by road infrastructure with considerable damage and lack of safety systems for drivers and pedestrians. The findings of this study demonstrate a similar situation with the concentration and high number of traffic accidents occurring in populated areas of other countries ^9^^,^^11^^,^^19^^,^^23^ with conditions quite similar to those observed in the SSMA.

From the geographical point of view, one of the main explanations focuses on population density, urban hierarchy index and vehicular flow as the main factors involved ^24^. In this study, the roads with hotspots coincide in some characteristics, such as population and vehicular density, as well as poor road safety.

There are five points in the SSMA where a large number of traffic accidents occur. The point of greatest interest is located to the southwest of SSMA and is identified as C4 in Figure 3. C4 is located on a primary road with high vehicular flow (Venezuela Boulevard) and one of the most important bus stations in El Salvador (West Terminal). Similar results in Brazil show that the points with the highest number of traffic accidents and injuries are located in the vicinity of a bus station ^25^. Bus stations are generally surrounded by a lot of commerce and pedestrians, and have been identified as frequent sites of traffic accidents ^23^. To this can be added to a large number of urban public transport vehicles and cabs, also identified as elements that can influence the increase in traffic accidents ^23^^,^^26^. This could be one of the main reasons for the high concentration of traffic accidents and injuries in C4. On the other hand, the high density of traffic accidents in the center, east and north of SSMA may be due to the high concentration of population and vehicles that has been increasing in the last decade ^5^^,^^15^. Also, unplanned growth, lack of land use planning ^5^^,^^8^^)^ and driver behavioral factors ^5^.

The observation count defined 12 areas with a number of traffic accidents and injuries greater than 45; however, the analysis of hotspots identified only five of these (C1, C4, C7, C10 and C11) as statistically significant. These five grids account for almost half of the traffic accidents in the SSMA, and coincide with the areas where the greatest number of injuries were recorded, with the exception of C7. According to our results, most injuries occurred on working days (Monday to Friday), a period in which there is also a greater flow of people entering from the cities considered dormitory cities and from other parts of the country for work, academic, business, medical care, among others. The median comparison analysis indicates that there is a difference between the number of traffic accidents that occur on weekdays, weekends and holidays. In El Salvador, as in other countries worldwide, work activity decreases during weekends and holidays. During these periods, people tend to leave the SSMA, which could be an explanation for the decrease in traffic accidents and injuries during these periods. However, no statistically significant difference was found between the number of injuries during weekends and holidays.

The results of spatial patterns and the data obtained from the areas with more accidents in the AMSS highlight the existence of narrow primary roads, deficient signaling, mainly that corresponding to the roads (horizontal signaling), damage to the roads, lack of containment systems, constant illegal bus stops on all roads and areas with the presence of many pedestrians without any type of road signaling. Other aspects such as cracks, repairs and potholes identified in C4, C7 and C10 could be caused, for example, by environmental conditions and the daily vehicular load, as suggested by Pérez-Fortes and Giudici (2022) ^27^. Drivers tend to avoid potholes at high speed, which can lead to loss of control of the vehicle, invasion of another lane, impact with other objects on the road, and pedestrian collisions. The streets in all the grids lacked pedestrian signals and vehicle containment systems, with the exception of C10, which has concrete and metal barriers, but these were partially damaged. In a model conducted to determine the severity of traffic accidents, land used for housing construction in urban areas, poor road signs and increased speed in urban areas with pedestrians were identified as the most influential variables ^13^. The lack of lighting observed in C10 may be a contributing factor to the increased risk of a traffic accident due to poor visibility on the roads ^26^, a problem that may be aggravated by the absence of traffic signs in C1, C7, and C10. Poor visibility and road signs are factors that contribute to the occurrence of traffic accidents ^28^^,^^29^. Besides, the large number of billboards in the main grids with the highest number of traffic accidents must also be considered. Billboards have been associated with traffic accidents due to the distraction they cause for drivers, as well as the risk that exists in the combination of billboards, high pedestrian traffic, and dense traffic ^30^.

Studies in which GIS have been used to analyze traffic accidents are limited in Central America; however, they have contributed to the understanding of the particular spatial patterns identified in some urban areas of the region. These studies are based on the specific identification of traffic accidents ^27^^,^^29^ and the application of a multivariate model to determine the frequency of traffic accidents based on factors that contribute to this problem ^31^. In El Salvador, the application of GIS has been useful to highlight issues of public health interest ^32^^,^^33^. However, it has not yet been used for the analysis of traffic accidents.

One of the most important limitations of this study is the lack of access to a database of people killed in traffic accidents, a variable that could increase the number of hotspots. On the other hand, the three-year gap between the occurrence of traffic accidents and the field inspection may not fully reflect the conditions at the time of the events. Finally, inaccuracy, errors in the georeferenced points and the non-use of field measurement equipment are other limitations to consider.

Future studies should include other variables (number of fatalities, type of vehicles, time of traffic accidents, causes of accidents, among others) that will help to further analyze this problem. Although the results do not have a direct geographic association with road safety factors, they do make it possible to determine the concentrations of traffic accidents and describe elements for first interventions and future association studies. Therefore, other analyses that contribute to a better approximation of the geographic context and the incidence of traffic accidents should be considered.

In conclusion, there are five areas in the SSMA with a large number of traffic accidents and injuries. They are characterized by road infrastructure with considerable damage, lack of safety systems for drivers and pedestrians, deficient road signs, visual pollution and lack of lighting. The geographic analysis of traffic accidents in the SSMA shows the usefulness of determining areas with higher occurrence. The findings of this study are a precedent of the behavior of traffic accidents in the city with the most traffic accidents in Latin America and may be useful for implementing improvements in surveillance and road safety controls.
