# Supplementary material for: Geographic areas with the highest concentration of traffic accidents in San Salvador, El Salvador: a spatial analysis of the 2014-2018 period
Source: Rev Peru Med Exp Salud Publica. 2023 Dec 18;40(4):413–22. doi: 10.17843/rpmesp.2023.404.12963 (PMC11138829; doi:10.17843/rpmesp.2023.404.12963)
Supplement: Supplementary material. — Available in the electronic version of the RPMESP. [file rpmesp-40-04-12963-s001.pdf]

Material Suplementario

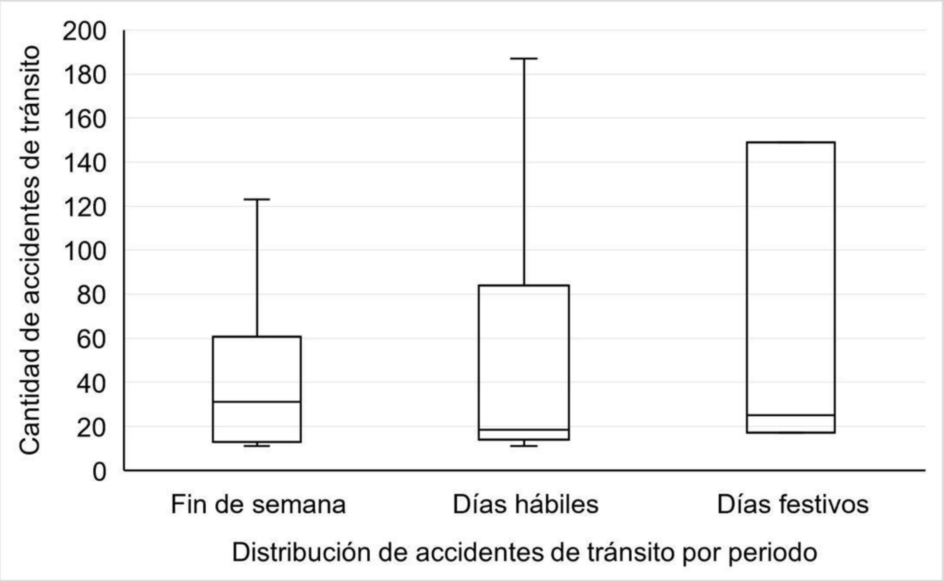

Material suplementario 1. Distribución de accidentes de tránsito por periodos en los puntos de mayor ocurrencia

Material suplementario 1. Características de seguridad vial de las zonas con mayor ocurrencia de accidentes de tránsito en el Área Metropolitana de San Salvador

**Aspectos observados en las vías**	**C1**	**C4**	**C7**	**C10**
**Infraestructura vial**				
**Tipo de vía**				
Primarias		X	X	X
Secundarias	X			
**Material de construcción de la vía**				
Asfalto	X	X		
Concreto			X	X
**Ancho total de la vía**				
14 m		X		
16 m	X			
24 m			X	
40 m				X
**Sistemas de contención de vehículos**				
Barra de concreto simple				X
Barra metálica				X
Ninguno	X	X	X	
**Ubicación del sistema de contención de vehículos**				
En el arcén				X
En medio				X
**Estado de los sistemas de contención de vehículos**				
Parcialmente dañado				X
**Daños de las vías**				
Baches		X	X	X

Corrugaciones		X		
Corrimientos		X		
Fisuras longitudinales		X		X
Fisuras transversales		X	X	X
Fisuras esquinadas				X
Fisuras múltiples				X
Pulimiento superficial		X	X	
Peladuras o desprendimiento		X	X	
Ninguno	X			
**Flujo vehicular**				
**Velocidad máxima permitida**				
40 km/h	X			
60 km/h		X	X	X
**Tipo de vehículos que circulan**				
Liviano particular	X	X	X	X
Pesado de carga		X	X	
Transporte colectivo		X	X	X
Motocicletas	X	X	X	X
Bicicletas	X	X	X	X
**Sentidos direccionales y otras características**				
Sentido único	X			
Doble sentido		X	X	X
Doble carril por lado	X	X	X	X
Intersecciones en +	X			
Intersecciones en Y		X	X	
Intersecciones en T		X	X	X
Vías planas		X		

Vías en pendientes		X		X
Curvas		X		X
Puentes		X		
Túneles		X		
Presencia de iluminación	X	X	X	
**Señalización vial**				
**Señalización vertical**				
Preventiva	X			X
Restrictiva	X			X
Informativa		X		X
Ninguna			X	
**Señalización horizontal**				
Línea de borde de pavimento		X		
Ninguna	X		X	X
**Seguridad peatonal**				
**Presencia de peatones**				
Alto flujo peatonal	X	X		
**Características de seguridad para peatones**				
Disponibilidad de aceras	X	X	X	X
Disponibilidad de rampas	X			
Disponibilidad de pasarelas		X		X
Disponibilidad de arcén				X
Zona de cruce peatonal	X	X	X	X
Zona con mucha afluencia de peatones	X	X	X	X
Existe cámara de monitoreo en el lugar	X	X	X	X
Falta de señalización para seguridad de peatones	X	X	X	X
**Ancho aproximado de pasarelas**				

1.5 m				X
1.7 m		X		
**Estado de las pasarelas**				
Buen estado		X		X
**Ancho aproximado de aceras**				-
1.5 m			X	
2 m	X	X		
**Estado de las aceras**				
Buen estado	X		X	
Parcialmente dañado		X		
**Ancho del arcén**				
1.5 m				X
**Estado del arcén**				
Parcialmente dañado				X
**Presencia de paradas de buses legales**				
Si		X		X
No	X		X	
**Contaminación visual y obstaculización en las vías**				
**Tipo de contaminación visual**				
Vallas publicitarias		X	X	
Señalización vial combinada con publicidad	X			
Presencia de vegetación				X
**Tipo de actividad presente en la zona**				
Zona de comercio formal	X	X	X	
Zona de comercio informal		X		
Zona industrial				X
Zona sin actividad económica				X

Zona recreativa	X			
Terminal de autobuses		X		
**Obstáculos**				
Utilización de aceras como estacionamiento	X	X	X	
Utilización de arcén como estacionamiento				X
Vehículos estacionados en la vía	X		X	
