# Supplementary material for: Geographic areas with the highest concentration of traffic accidents in San Salvador, El Salvador: a spatial analysis of the 2014-2018 period
Source: Rev Peru Med Exp Salud Publica. 2023 Dec 18;40(4):413–22. doi: 10.17843/rpmesp.2023.404.12963 (PMC11138829; doi:10.17843/rpmesp.2023.404.12963)
Supplement: Translation

# Translation: Áreas geográficas con mayor concentración de accidentes de tránsito en San Salvador, El Salvador: un análisis espacial del periodo 2014-2018

**Authors:** Roberto Mejía, Edgar Quinteros, Alexandre Ribó Arnau

**Affiliations:** 1 Instituto Nacional de Salud, San Salvador, El Salvador.; 2 Departament d'ensenyament, Generalitat de Catalunya, Spain.

**Keywords:** Accidentes de Tránsito, Prevención de Accidentes, Sistemas de Información Geográfica (SIG), El Salvador, Centroamérica

## Abstract

**Objetivo.:**

Identificar las áreas de mayor concentración de accidentes de tránsito y lesionados en el Área Metropolitana de San Salvador (AMSS).

**Materiales y métodos.:**

Los accidentes de tránsito se analizaron espacialmente mediante la ubicación puntual y por la sumatoria de eventos en áreas de 200 m^2^. La ubicación puntual se analizó mediante «análisis de vecinos más cercanos», mientras que las áreas con la sumatoria de accidentes de tránsito se analizaron mediante Gi* de Getis-Ord para obtener los puntos calientes. Los puntos calientes resultantes con mayor concentración de accidentes de tránsito en el AMSS se evaluaron en campo mediante un formulario de observación de las características de infraestructura y seguridad vial.

**Resultados.:**

Al analizar 8191 accidentes de tránsito reportados entre 2014‒2018, se identificaron cinco áreas con mayor cantidad de accidentes de tránsito y lesionados, principalmente sobre vías primarias.

**Conclusión.:**

Los sitios de mayor concentración de accidentes de tránsito y lesionados se caracterizan por una infraestructura vial con daños considerables y falta de sistemas de seguridad para conductores y peatones. El análisis espacial de los accidentes de tránsito y lesionados puede contribuir a mejorar la vigilancia y seguridad vial en el AMSS.

## INTRODUCCIÓN

Los accidentes de tránsito son un problema grave de salud pública a nivel mundial. De acuerdo con la Organización Mundial de la Salud (OMS), las personas fallecidas por accidentes de tránsito aumentaron de 1,15 a 1,35 millones entre el 2000 y 2016 ^1^. En América Latina, en el 2016 se reportaron 154 997 fallecidos que representaron el 11% de las muertes a nivel mundial ^2^. Los países de América Latina más afectados por los accidentes de tránsito son El Salvador, Brasil, Venezuela, Colombia y Ecuador ^3^^,^^4^.

En El Salvador, los accidentes de tránsito representan un problema importante de salud pública. En el periodo 2014‒2018 se reportaron 38 531 lesionados y un total de 3573 víctimas fatales ^5^. Cada año se invierte un promedio de 15 millones de dólares en atención a víctimas de accidentes de tránsito ^6^. En la ciudad de San Salvador donde se reporta el mayor número de accidentes de tránsito en América Latina ^3^ se ubica gran parte del Área Metropolitana de San Salvador (AMSS). Durante el 2019, el 51,5 % de los accidentes de tránsito del país ocurrieron en el AMSS ^5^.

Algunas de las vías del AMSS han sido construidas de forma no planificada y bajo ciertas condiciones sociales que determinaron su actual diseño y condición. El AMSS cuenta con pequeñas partes de asentamientos urbanos aislados que carecen de infraestructura y servicios básicos. Estos asentamientos iniciaron desde mediados de los años 50 como producto del crecimiento no planificado y la falta de una ley de ordenamiento territorial ^7^^,^^8^. A esto se sumó el flujo migratorio ocurrido en la década de los años 80 desde el interior del país hacia el AMSS, como producto del conflicto armado en El Salvador ^7^.

El análisis geográfico constituye una de varias alternativas de análisis para comprender el problema de los accidentes de tránsito. Su contribución se basa en identificar patrones de distribución espacial de los accidentes de tránsito y los factores del entorno que puedan estar relacionados. En las últimas décadas, se ha hecho uso de los Sistemas de Información Geográfica (SIG) para analizar los patrones de distribución espacial de puntos críticos de accidentes de tránsito en áreas urbanas ^9^^,^^10^. Los SIG, un conjunto de herramientas y componentes que permiten gestionar información mediante el uso de tecnología espacial, han sido ampliamente utilizados en salud pública. Particularmente, en América Latina, han sido útiles en identificar la distribución espacial de las lesiones y mortalidad por accidentes de tránsito en áreas de gran circulación vehicular ^11^^,^^12^. En otros estudios también se ha estimado la vulnerabilidad de usuarios en las vías a partir del análisis de densidad espacial de colisiones y características de estas para la ocurrencia de accidentes de tránsito ^13^^,^^14^.

En El Salvador hay una integración cada vez más creciente de información geoespacial en las bases de datos de vigilancia en salud, pero aún no se dispone de estudios geográficos sobre áreas de mayor concurrencia de accidentes de tránsito. El objetivo de este estudio es identificar las áreas geográficas de mayor concentración de accidentes de tránsito y lesionados en el AMSS en el periodo de 2014‒2018. Los resultados contribuyen al conocimiento de los patrones territoriales con mayor accidentabilidad vial mediante la aplicación de herramientas de análisis disponibles en los SIG.

**MENSAJE CLAVE**

Motivación para realizar el estudio: Los accidentes de tránsito son problema grave de salud pública en El Salvador, en especial en el Área Metropolitana de San Salvador (AMSS). No hay evidencia científica de áreas críticas en el AMSS y el país.

Principales hallazgos: Se identificaron cinco áreas geográficas en las que se registraron una gran cantidad de accidentes de tránsito y lesionados. Se caracterizan por presentar una infraestructura vial con daños considerables, falta de sistemas de seguridad, señalización vial deficiente, contaminación visual y falta de iluminación.

Implicancias: La identificación de áreas críticas de accidentes de tránsito puede contribuir a la creación de políticas públicas para la prevención.

## MATERIALES Y MÉTODOS

### Diseño de estudio

Este es un estudio de tipo transversal analítico con información de las atenciones de accidentes de tránsito y lesionados en el AMSS. La información proviene del Sistema de Emergencias Médicas (SEM), una red de coordinación de atención de emergencias a nivel nacional del Ministerio de Salud (MINSAL) que cuenta con un sistema de respuesta inmediata.

### Área de estudio

El AMSS es una conurbación situada sobre 14 municipios (Figura 1), con una extensión territorial de 610,84 km^2^, una población de 1 693 186 habitantes y una densidad de población de 2772 h/km^2 (^^15^.

**Figura 1:** Ubicación del AMSS y distribución espacial de los accidentes de tránsito durante 2014-2018.

### Datos

Los datos de accidentes de tránsito y lesionados ocurridos en el AMSS corresponden al periodo 2014-2018. Los datos y permisos de uso fueron solicitados de forma escrita al SEM quienes enviaron un archivo Excel. El registro del SEM no incluye los casos de personas fallecidas a causa de accidentes de tránsito, estos casos son atendidos por la Policía Nacional Civil e Instituto de Medicina Legal, por lo tanto, en este estudio no se han analizado los fallecidos por accidentes de tránsito.

Los 15 497 registros proporcionados por SEM corresponden a las atenciones en los 262 municipios del país. Para fines de este estudio, se seleccionaron únicamente 8191 accidentes de tránsito ocurridos en el AMSS. La información sobre la ubicación espacial donde se produjeron los accidentes de tránsito fue obtenida mediante el sistema de posicionamiento global (GPS, por sus siglas en inglés) o manualmente utilizando mapas web. Debido a errores de ubicación que presentaban las coordenadas, fue necesario realizar una adecuación mediante la verificación de direcciones postales del registro de atención del SEM y el uso de la herramienta de movimiento de objetos espaciales disponible en los SIG.

### Geocodificación de datos

Para el análisis espacial, previamente fue necesario realizar un proceso de geocodificación, que consistió en transformar las variables de estudio en ubicaciones geográficas. A partir de los datos del SEM se creó una capa de puntos de los accidentes de tránsito ocurridos en el AMSS. El análisis también requirió de la utilización de cartografía referente a carreteras y delimitación geográfica nacional que fue obtenida del Centro Nacional de Registros. Utilizando la capa de carreteras, se creó una nueva selección únicamente de las vías que corresponden al AMSS. Finalmente, con la extensión territorial del AMSS se creó una malla de cuadrículas de 200 m^2^ que contenía la sumatoria de accidentes de tránsito, una técnica de mapeo conocida como conteo por observación, similar al utilizada por Adams *et al*. ^16^.

### Variables de estudio

Para fines del análisis de este estudio, se realizó una selección y reclasificación de las variables que originalmente contenía la información del SEM. Se eliminaron las variables con información repetida, con errores de digitación o con campos vacíos. Las variables «Año», «Día de semana», «Fin de semana» y «Días festivos» fueron reclasificadas a partir de la información de la variable «fechadedemanda». Las variables «Colisión» y «Choque» se definieron a partir de la información de la variable «tipodedemanda». Finalmente, las variables reclasificadas como «lesionados», «motociclistas» y «atropellados» fueron obtenidas de la variable «motivoliteral» que contenía detalles del tipo de vehículo, lesionados que iban a bordo del vehículo o atropellados en el accidente de tránsito. En la tabla suplementaria 1 se muestra la descripción de las variables de estudio.

### Inspección de sitios de mayor concentración de accidentes en el AMSS

Se realizó una visita a las áreas identificadas como puntos calientes, con el fin de observar la infraestructura de la vía y aspectos de seguridad vial. Las visitas de campo fueron realizadas por los investigadores del estudio, con apoyo de médicos residentes de epidemiología. Toda la información observada se recolectó a través de un formulario de creación propia en *KoBo ToolBox*^17^, formulario disponible en https://ee.kobotoolbox.org/eTC7EiC2. Algunos términos utilizados sobre características del sistema vial se basaron en lo establecido en la Ley de Transporte de El Salvador, artículos científicos y terminología definida por organismos internacionales ^18^^-^^20^. Para la validación del formulario, los autores de este estudio realizaron una prueba piloto en tres puntos críticos a las afueras del AMSS en un día de semana durante horas de mayor concurrencia vehicular.

### Análisis estadístico

Se realizó un análisis descriptivo para identificar la frecuencia de los accidentes de tránsito, la cantidad de personas lesionadas, el tipo de colisión, la distribución por mes y año, y características de infraestructura de seguridad vial de los sitios con mayor concentración de accidentes de tránsito. Se comprobó la distribución de la población mediante la prueba Kolmogorov-Smirnov y una comparación de medianas de la cantidad de accidentes y lesionados durante los días hábiles (lunes a viernes), fines de semana (sábado y domingo) y días festivos (23 días en el año) a través de la prueba Kruskal Wallis.

### Análisis geográfico

Para análisis de los datos y diseño de visualización se utilizó el *software*, ArcGIS Desktop versión 10.1 y QGIS versión 3,16, respectivamente. El análisis geográfico de los accidentes de tránsito se hizo a nivel puntual y por áreas. A nivel puntual se identificó la distribución de accidentes de tránsito en la red vial utilizando el método no paramétrico denominado Análisis de Vecinos más Cercanos ^21^^)^ para medir la distancia promedio entre cada punto.

A nivel de áreas, se determinaron puntos calientes de accidentes de tránsito a partir de polígonos de 200 m^2^. Se utilizó la herramienta estadística Gi* de Getis-Ord ^22^^)^ para obtener la significancia estadística de cada cuadrícula. Los valores resultantes del análisis mediante Gi* de Getis-Ord muestra puntos calientes con valores altos o bajos de accidentes de tránsito. El análisis de los puntos calientes puede presentar tres resultados, agrupamiento alto, medio o bajo. Para cada uno de estos agrupamientos, el software presenta valores diferentes de puntuación Z (valores de desviación estándar), de p y de confianza. Los valores de puntuación Z > 1,65 con una p < 0,1 presentan una confianza del 90 % (agrupamiento bajo); los valores de puntuación Z > 1,96 con una p < 0,05 presentan un valor de confianza de 95 % (agrupamiento medio) y los valores de puntuación Z >2,58 con una p < 0,01 presentan un valor de confianza del 99 % (agrupamiento alto). En la Figura 2 se presenta un diagrama de flujo del procesamiento de los datos y análisis de accidentes de tránsito en el AMSS.

**Figura 2:** Diagrama de flujo del procesamiento de datos y análisis de los accidentes de tránsito en el Área Metropolitana de San Salvador.

### Aspectos éticos

Este estudio no implicó el análisis de muestras provenientes de humanos, sin embargo, fue revisado y aprobado por el Comité de Ética del Instituto Nacional de Salud. La información se trabajó de forma confidencial entre los autores de este estudio, respetando todos los aspectos referentes a buenas prácticas clínicas y ética en investigación.

## RESULTADOS

### Análisis estadístico

En total se registraron 8191 accidentes de tránsito, en los que resultaron lesionadas 8996 personas, de las cuales el 75,6% son hombres. La edad de los afectados varía de 0 a 98 años, con un promedio de 33,6 años (desviación estándar 14,4). En más del 60% de los accidentes de tránsito estuvieron involucradas personas entre los 20 y 39 años. En el 30,5% de los accidentes de tránsito estuvo involucrado al menos un motociclista. En el 40,9% de los accidentes de tránsito se atropelló como mínimo a una persona. El 8,7% de los accidentes de tránsito fue provocado por un choque o colisión. La tasa de incidencia acumulada para el periodo en estudio es de 531,3 × 100 000 habitantes.

### Análisis geográfico

*Patrón de distribución de accidentes de tránsito*

El «análisis de vecinos más cercanos» identificó un patrón de puntos agrupados, con un índice del vecino más cercano promedio de 0,13, una distancia promedio observada de 17,40 m y una distancia promedio esperada de 130,90 m. El mayor número de accidentes de tránsito y lesionados se concentra en 12 cuadrículas identificadas en la figura 3 con un código (una letra C y un número). En el área de la C4 se registró la mayor cantidad de accidentes de tránsito (2895) y de lesionados (3210). En segundo lugar, en la C11 se registraron 474 accidentes de tránsito y 538 lesionados. En tercer lugar, se encuentra la C1, ubicada en el oeste del AMSS, donde se reportaron 297 accidentes de tránsito y 340 lesionados. En la mayoría de las cuadrículas ocurrieron entre 1 y 15 accidentes de tránsito. 

**Figura 3:** Conteo de accidentes de tránsito en el Área Metropolitana de San Salvador por cada 200 m².

*Puntos calientes de accidentes de tránsito*

En la Figura 4 se puede observar los resultados obtenidos a partir del análisis de puntos calientes. Se identificó un total de cinco puntos calientes que suman un total de 4031 accidentes de tránsito en el que resultaron 4483 lesionados durante el periodo de estudio. La cuadrícula con mayor densidad es la C4 con un valor de puntuación Z de 31,3 ( p <0,001). La segunda cuadrícula con una densidad alta de accidentes de tránsito es la C11 (puntuación Z 5,06; p <0,001), seguida por la C1 (puntuación Z 3,09; p <0,001), C7 (puntuación Z de 1,98; p=0,04) y finalmente la C10 con una puntuación Z de 1,83; p= 0,06.

**Figura 4:** Puntos calientes de accidentes de tránsito en el Área Metropolitana de San Salvador.

*Distribución espacial de accidentes de tránsito y lesionados según días hábiles, fin de semana o días festivos*

Durante los días hábiles (figura 5a) se registró una cantidad mayor de accidentes de tránsito (5626) en comparación con los fines de semana (2174) (figura 5b) y días festivos (391) (figura 5c). Los accidentes de tránsito registrados en días hábiles están distribuidos en 1542 puntos ubicados hacia el noroeste del AMSS. En los días hábiles se registraron 56 puntos en los que ocurrieron 11 o más accidentes de tránsito (mediana=18, Q3=84, Q1=14). Durante los fines de semana, en 28 puntos ocurrieron 11 o más accidentes de tránsito (mediana=31, Q3=61, Q1=13) y en días festivos se registraron 3 puntos en los que ocurrieron 11 o más accidentes de tránsito (mediana=25, Q3=149, Q1=17) (Material suplementario 1). A través de la comparación de medianas se identificó que existe una diferencia significativa (p<0,01) entre la cantidad de accidentes de tránsito que ocurren en días hábiles, fines de semana y días festivos.

**Figura 5:** Distribución espacial de accidentes de tránsito durante días hábiles (5a), fines de semana (5b) y días festivos (5c)

Durante los días hábiles se registró un total de 6098 lesionados, con una mediana de 4,69. Los fines de semana se reportaron 2457 lesionados con una mediana diaria de 5,01 lesionados por fin de semana. En los accidentes de tránsito ocurridos en los días festivos, resultaron lesionadas 441 personas, con un promedio diario de 3,83 lesionados. Sin embargo, a través de un análisis de comparación de medianas, se comprobó que existe diferencia estadísticamente significativa (p<0,01) entre la cantidad de personas que resultan lesionadas en los accidentes de tránsito ocurridos en los días hábiles, fines de semana y días festivos.

### Caracterización de los sitios de mayor ocurrencia de accidentes de tránsito

En el material suplementario 2 muestra información *in situ* de las características viales de las cuadrículas con significancia estadística (puntos calientes) en el AMSS. La C11 fue inspeccionada debido a la inseguridad social de la zona. Tres de las cuadrículas (C4, C7 y C10) tienen en su mayoría vías primarias, en la C1 y C4 las vías están construidas a base de asfalto y en la C10 y C7 a base de cemento. Las vías más anchas se encuentran en la C12 (24 m) y C10 (40 m). La C1 y C7 tienen vías más angostas con 14 m y 16 m, respectivamente.

La C1 es la única área que no presenta daños estructurales y solamente la vía de la C10 tiene sistema de contención de vehículos, pero está parcialmente dañado. En cuanto a la señalización vial, predomina la señalización vial vertical de tipo preventiva (señales de alto), sin embargo, esta se encuentra en mal estado. En tres de las cuadrículas (C4, C7, C10) existen paradas de buses ilegales, y en las C1, C4 y C7 existe una gran cantidad de vallas publicitarias. En todas las cuadrículas se observaron zonas económicamente activas. En las cuadrículas C1, C4 y C7 hay infraestructura para peatones, como aceras, la C10 cuenta con arcén y la C4 y C10 cuentan con pasarelas.

En la C4 se encuentra ubicada una de las principales estaciones de buses de la capital, con alto flujo peatonal y vehicular. En los alrededores de la estación de buses las aceras son utilizadas por el comercio informal y como estacionamiento de vehículos. Además, algunos vehículos se encontraban estacionados en la vía, reduciendo la circulación vehicular a un carril por sentido.

## DISCUSIÓN

Este estudio ha identificado cinco puntos calientes donde se concentra la mayor cantidad de accidentes de tránsito y lesionados en el AMSS. Estos puntos calientes se caracterizan por una infraestructura vial con daños considerables y falta de sistemas de seguridad para conductores y peatones. Los hallazgos de este estudio demuestran una situación similar con la concentración y alto número de accidentes de tránsito ocurridos en áreas pobladas de otros países ^9^^,^^11^^,^^19^^,^^23^ con condiciones bastantes similares a las observadas en el AMSS.

Desde el punto geográfico, una de las principales explicaciones se centra en la densidad de población, índice de jerarquía urbana y flujo vehicular como principales factores involucrados ^24^. Las vías que presentan puntos calientes en este estudio coinciden en algunas características, como la densidad de población y vehicular, así como la poca seguridad vial.

En el AMSS se ubican cinco puntos en los cuales ocurre una gran cantidad de accidentes de tránsito. El punto de mayor interés se encuentra ubicado al suroeste del AMSS identificado como C4 en la Figura 3. En este punto se reportan cifras de accidentes de tránsito y lesionados muy superiores a las identificadas en otras cuadrículas. La C4 se ubica en una vía primaria con alto flujo vehicular (Bulevar Venezuela) y una de las estaciones de buses más importantes de El Salvador (Terminal de Occidente). Resultados similares en Brasil, evidencian que los puntos con mayor cantidad de accidente de tránsito y personas lesionadas se encuentran en las cercanías de una estación de autobuses ^25^. Las estaciones de autobuses generalmente están rodeadas de mucho comercio, presencia de peatones y se han identificado como sitios frecuentes de accidentes de tránsito ^23^. A esto se puede sumar una gran cantidad de vehículos de transporte público urbano y taxis, también identificados como elementos que pueden influir en el incremento de accidentes de tránsito ^23^^,^^26^. Lo anterior, podría ser una de las principales razones de la alta concentración de accidentes de tránsito y lesionados en la C4. Por otro lado, la alta densidad de accidentes de tránsito en el centro, este y norte del AMSS, puede deberse a la alta concentración de población y vehículos que ha venido incrementando en la última década ^5^^,^^15^. Así mismo, el crecimiento no planificado, falta de ordenamiento territorial ^5^^,^^8^ y factores comportamentales de los conductores ^5^.

El conteo por observación define 12 áreas con un número de accidentes de tránsito y lesionados mayor de 45, sin embargo, con el análisis de puntos calientes solamente se identificó a cinco de estas (C1, C4, C7, C10 y C11) con significancia estadística. En estas cinco cuadrículas se concentra casi la mitad de los accidentes de tránsito del AMSS, y coinciden con las áreas donde se registró la mayor cantidad de lesionados, a excepción de la C7. De acuerdo con los resultados, la concentración de lesionados es mayor en días hábiles (lunes a viernes), periodo en el que también hay un mayor flujo de personas que ingresan desde las ciudades consideradas dormitorio y de otras partes del país por motivos laborales, académicos, negocios, atención médica, entre otras. El análisis de comparación de medianas, indica que, existe diferencia entre la cantidad de accidentes de tránsito que ocurren en los días de semana, fines de semana y días festivos. En El Salvador, como en otros países a nivel mundial, la actividad laboral disminuye durante los fines de semana y días festivos. Durante esos periodos, las personas acostumbran a salir del AMSS, esta podría ser una explicación de la disminución de accidentes de tránsito y lesionados durante estos periodos. Sin embargo, no se encontró diferencia estadísticamente significativa entre la cantidad de lesionados durante los fines de semana y los días festivos.

Los resultados de patrones espaciales y los datos obtenidos en las áreas con más accidentes en el AMSS, destacan la existencia de vías primarias angostas, señalización deficiente, principalmente la que corresponde a las vías (señalización horizontal), daños en las vías, falta de sistemas de contención, paradas de buses ilegales constantes en todas las vías y zonas con presencia de muchos peatones sin ningún tipo de señalización vial. Otros aspectos como fisuras, reparaciones y baches identificados en de las C4, C7 y C10, podrían ser provocados, por ejemplo, por condiciones propias del ambiente y de la carga vehicular diaria, como sugiere Pérez-Fortes y Giudici (2022) ^27^. Los conductores tienden a evadir los baches a una gran velocidad, lo que puede provocar la pérdida del control del vehículo, invasión de otro carril, impacto con otros objetos de la vía y atropello de peatones. Las calles de todas las cuadrículas analizadas carecen de señalización para peatones y de sistemas de contención de vehículos, a excepción de la C10 que cuenta con barreras de cemento y metal, pero que se encuentran parcialmente dañadas. En un modelo realizado para determinar la gravedad de los accidentes de tránsito, se identificó el uso del suelo utilizado para la construcción de viviendas en zonas urbanas, señalización vial deficiente y aumento de velocidad en zonas urbanas con presencia de peatones como variables de mayor influencia ^13^. La falta de iluminación observada en la C10 puede ser un factor que contribuya al incremento del riesgo de sufrir un accidente de tránsito por la poca visibilidad en las carreteras ^26^, un problema que puede agravarse con la ausencia de señales de tránsito observada en las C1, C7 y C10. La poca visibilidad y la señalización en las carreteras son factores que contribuyen a la ocurrencia de accidentes de tránsito ^28^^,^^29^. A esto también debe agregarse la gran cantidad de vallas publicitarias observadas en las principales cuadrículas con mayor cantidad de accidentes de tránsito. Las vallas publicitarias han sido asociadas con la ocurrencia de accidentes de tránsito debido a la distracción que generan para los conductores, así como el riesgo existente en la combinación de vallas publicitarias, alta afluencia de peatones y tráfico denso ^30^.

En Centroamérica, los estudios en los que se ha aplicado los SIG para analizar los accidentes de tránsito son limitados, sin embargo, han contribuido en la comprensión de los patrones espaciales particulares identificados en algunas zonas urbanas de la región. Estos estudios se basan en la identificación puntual de accidentes de tránsito ^27^^,^^29^ y la aplicación de un modelo multivariado para determinar la frecuencia de accidentes de tránsito a partir de factores que contribuyen a esta problemática ^31^. En El Salvador, la aplicación de los SIG ha sido útil para evidenciar temas de interés de salud pública ^32^^,^^33^. Sin embargo, aún no es utilizado para el análisis de los accidentes de tránsito.

Entre las limitantes más importantes de este estudio se encuentra la falta de acceso a una base de datos de personas fallecidas en accidentes de tránsito, una variable que podría incrementar el número de puntos calientes. Por otra parte, la diferencia de tres años entre la ocurrencia de los accidentes de tránsito y la inspección realizada en campo podría no reflejar completamente las condiciones al momento en que se produjeron los eventos. Finalmente, la imprecisión, errores en los puntos georreferenciados y la no utilización de equipos de medición en campo, son otras limitantes que considerar.

Futuros estudios deben integrar otras variables (número de fallecidos, tipo de vehículos, hora de los accidentes de tránsito, causas del accidente, entre otras) que ayuden a profundizar más el análisis de este problema. Aunque los resultados no tienen un alcance de asociación geográfica directa con factores de seguridad vial, si permite determinar las concentraciones de accidentes de tránsito y describir elementos para primeras intervenciones y futuros estudios de asociación. Por tanto, deben considerarse otros análisis que contribuyan a una mejor aproximación del contexto geográfico y la incidencia de accidentes de tránsito.

En conclusión, en el AMSS existen cinco áreas en las que se registra una gran cantidad de accidentes de tránsito y lesionados. Se caracterizan por presentar una infraestructura vial con daños considerables, falta de sistemas de seguridad para conductores y peatones, señalización vial deficiente, contaminación visual y falta de iluminación. El análisis geográfico de los accidentes de tránsito en el AMSS muestra la utilidad para determinar áreas con mayor ocurrencia. Los hallazgos de este estudio son un precedente del comportamiento de los accidentes de tránsito en la ciudad con más accidentes de tránsito en América Latina y pueden ser de utilidad para implementar mejoras en los controles de vigilancia y de seguridad vial.
